# Multi-parametric quantitative MRI of the lower limb muscles in a longitudinal study of limb-girdle muscular dystrophy R9

**DOI:** 10.1371/journal.pone.0321463

**Published:** 2025-04-28

**Authors:** Susanne S. Rauh, Pierre-Yves Baudin, Tanya Stojkovic, Simone Birnbaum, Valérie Decostre, Rachida-Lydie Zanfongnon, Yves Fromes, Melissa T. Hooijmans, Gustav J. Strijkers, Jean-Yves Hogrel, Sophie Olivier, Benjamin Marty, Harmen Reyngoudt

**Affiliations:** 1 Institute of Myology, Neuromuscular Investigation Center, NMR Laboratory, Paris, France; 2 Amsterdam Movement Sciences, Musculoskeletal Health, Amsterdam, The Netherlands; 3 Department of Biomedical Engineering and Physics, Amsterdam University Medical Center, University of Amsterdam, Amsterdam, The Netherlands; 4 Neuromuscular Reference Center, Institute of Myology, Pitié-Salpêtrière Hospital (AP-HP), Paris, France; 5 Institute of Myology, Neuromuscular Investigation Center, Neuromuscular Physiology and Evaluation Laboratory, Paris, France; 6 Généthon, Evry, France; 7 Vrije Universiteit Amsterdam, Amsterdam, The Netherlands; 8 Department of Radiology and Nuclear Medicine, Amsterdam University Medical Center, University of Amsterdam, Amsterdam, The Netherlands; 9 Atamyo Therapeutics, Evry, France; University of Miami Miller School of Medicine: University of Miami School of Medicine, UNITED STATES OF AMERICA

## Abstract

**Background and Objectives:**

Limb-girdle muscular dystrophy R9 (LGMD-R9) is a rare neuromuscular disease with no curative treatment. Sensitive non-invasive biomarkers are necessary to monitor disease progression and evaluate the efficacy of novel therapies. Here, we investigated several quantitative MRI parameters as suitable biomarkers for evaluating disease progression in LGMD-R9.

**Methods:**

Bilateral quantitative MRI of the lower limbs was performed in individuals with LGMD-R9 and healthy controls. Quantitative thigh and leg muscle MRI, functional tests (including time-up-and-go (TUG) and time-to-climb-4-stairs (4S climb)), and muscle strength tests were performed in individuals with LGMD-R9 at baseline, 1-year, and 2-years. qMRI included assessment of muscle fat fraction (FF), water T2, water T1, intramuscular pH from ^1^H MR spectroscopy, and diffusion tensor imaging (DTI) parameters. Differences between LGMD-R9 and controls, over time, and the relationship between baseline water T1 and water T2 parameters and disease progression (FF, functional and strength parameters) were assessed by linear mixed models and correlation analyses.

**Results:**

18 individuals with LGMD-R9 and 13 controls were enrolled. At baseline, elevated FF, water T2, water T1, and pH were observed in LGMD-R9 (p < 0.05). No differences between controls and LGMD-R9 were found in the DTI parameters. An overall tendency to an increase in FF and a decrease in functional measures were observed over 2 years. However, the changes did not reach significance (p = 0.057–0.752). Baseline water T1 and baseline water T2 correlated with the increase in FF (ΔFF) and change in TUG (ΔTUG) and 4S climb (Δ4S climb) over 2 years (correlation coefficient ≥  0.6, p < 0.05). No correlation with the strength measures was found.

**Conclusion:**

Our findings suggest that FF, water T2, water T1, and pH are effective biomarkers for LGMD-R9. The correlation of water T2 and water T1 with ΔFF, ΔTUG, and Δ4S climb suggests their potential in predicting disease progression.

## Introduction

Limb-girdle muscular dystrophies (LGMDs) are a group of rare hereditary neuromuscular disorders characterized by the replacement of muscle tissue by fat and progressive muscle weakness, starting at the proximal limbs [1, 2]. FKRP-related LGMD, or LGMD-R9, is caused by a mutation in the Fukutin-related protein (FKRP) gene, which encodes the FKRP protein involved in the glycosylation of α-dystroglycan. A loss of glycosylation of α-dystroglycan results in poor linkage of myocytes to the extracellular matrix and causes membrane fragility [3–5]. This ultimately leads to a loss of muscle fibers and strength and the replacement of muscle tissue by fat [6]. Typically, symptoms start around 10–20 years of age and include weakness of the proximal legs, spreading gradually to the distal leg muscles and the proximal upper limb, and are often accompanied by respiratory and/oras cardiac impairments [7]. This can cause exercise intolerance as well as lifestyle and functional impairments [6]. LGMD-R9 can present with different genotypes, whereby the homozygous c.826C >  A (L276I) mutation is most common [1]. There is currently no curative treatment for LGMD-R9; however, the development of novel treatment approaches is a topic of current research [8,9]. For the evaluation of therapies and to monitor individual disease progression rates, an understanding of the natural history of LGMD-R9 is crucial. Thus, there is a need for non-invasive, objective biomarkers to quantify disease progression.

Currently, functional testing is the primary non-invasive outcome measure for clinical trial endpoints and disease progression evaluation in neuromuscular diseases [10,11]. However, it has recently been shown that quantitative magnetic resonance imaging (qMRI) biomarkers are more sensitive to subtle changes over time than functional testing and less dependent on patient motivation [12,13]. The most frequently used qMRI biomarkers to assess neuromuscular disorders are muscle (contractile) cross-sectional area ((c)CSA), fat fraction (FF), and water T2 mapping [14,15]. CSA, cCSA, and FF are sensitive to disease progression and FF can depict changes over one year in various neuromuscular diseases [13,16–19]. However, the fat replacement of muscle tissue is irreversible. Sensitive biomarkers that can capture abnormalities and disease activity prior to muscle-fat replacement or that can predict disease progression would be of significant clinical value. While previous MRI studies on LGMD-R9 focused solely on FF and CSA as qMRI biomarkers [12,13,20,21], studies in other neuromuscular diseases have shown alterations in water T2 [22], water T1 [23], DTI parameters [24], and intramuscular pH [25] prior to muscle-fat replacement. Water T2 mapping and water T1 mapping are sensitive to disease activity and can depict deviations before muscle-fat replacement in neuromuscular diseases like Duchenne muscular dystrophy [22,25]. Although water T2 and water T1 are sensitive markers, they are influenced by various underlying pathophysiological processes such as necrosis, inflammation, edema, exercise, cell damage, denervation, and tumors, rendering them relatively non-specific [26,27]. DTI parameters and intramuscular pH may serve as more specific biomarkers of disease activity. DTI measures the movement of water molecules within tissue, with cell membranes acting as barriers to free diffusion. Consequently, alterations in muscle microstructure result in changes in diffusion patterns. Thus, DTI is sensitive to microstructural changes, potentially detecting these changes in muscle tissue prior to fat replacement. Muscle pH, measurable by ^1^H MR spectroscopy (^1^H MRS) through the pH-dependent chemical shift of carnosine relative to the water resonance [28], provides insights into potential alterations in ionic homeostasis within muscle tissue. Since carnosine is exclusively found intracellularly in healthy muscle, ^1^H MRS-derived pH accurately reflects intramuscular pH. Changes in pH values can indicate the presence of disease and cell membrane or tissue damage [29].

These qMRI biomarkers have the potential to predict disease progression in individuals and assist in selecting optimal candidates for clinical trials. Given that LGMD-R9 typically exhibits a mild phenotype [30], detecting changes in function or FF over short periods, such as one year, can be challenging [13]. Therefore, exploring qMRI biomarkers sensitive to disease activity holds significant promise for advancing the understanding and management of LGMD-R9.

The aim of this study was to investigate qMRI biomarkers for LGMD-R9 to discriminate between healthy and diseased muscle tissue and to explore their ability to predict disease progression in a two-year natural history study. Therefore, we used a multi-modal approach and assessed muscle FF and contractile CSA, and additionally added water T2, water T1, intramuscular pH, and DTI parameters at multiple diffusion times as well as functional and strength outcome measures.

## Methods

### Participants

The current study involved an analysis of a sub-cohort from the GNT-015-FKRP natural history study on LGMD-R9 (NCT03842878). This sub-cohort concerned a group of 18 participants enrolled at the French clinical site, recruited between 20.02.2020 and 13.12.2021, where a full quantitative lower limb muscle MRI protocol (including water T1 mapping, diffusion MRI, and ^1^H MRS) was performed. The analysis of FF and water T2 mapping of the entire natural history study of 52 participants conducted in France, Denmark, and the UK is subject to a separate publication. Inclusion criteria in the GNT-015-FKRP study were: 1) ≥  16 years at inclusion, 2) genetically confirmed diagnosis of LGMD-R9, and 3) ambulant, defined as being able to walk 10 meters within 30 seconds. Participants were excluded from the study if they had MRI contraindications.

Additionally, we have added a group of 13 healthy control participants, defined as having no history or diagnosis of neuromuscular disease and no muscle injury (recruitment period 10.03.2023–19.04.2023), not part of the GNT-015-FKRP study, with a similar age distribution and body mass index (BMI). Healthy control participants were scanned as part of a methodology MRI/S protocol approved by the local ethics committee (CPP-Ile de France VI—Groupe Hospitalier Pitié-Salpêtrière, ID RCB: 2012-A01689-34). Written informed consent was obtained from all participants to the GNT-015-FKRP study and the healthy control participants, adhering to the 1964 Declaration of Helsinki and its subsequent amendments.

### MRI protocol

MRI acquisitions of the lower extremities were carried out on a 3-T Prisma^Fit^ clinical system (Siemens Healthineers, Erlangen, Germany). Participants were positioned feet first supine. In the LGMD-R9 cohort three MRI exams were performed: at baseline, after 1-year, and after 2-years. In healthy controls, only one exam was performed.

Quantitative FF mapping, water T2 mapping, and water T1 mapping were performed in both thighs and legs using an 18-channel body radiofrequency (RF) coil in combination with a 32-channel posterior RF coil (schematic overview in S1 Fig in S1 Data). Matching positions between baseline, 1-year, and 2-year follow-ups were ensured by using anatomical landmarks for positioning of the center slice. For the thighs, the center slice was placed at the origin of the *adductor magnus* muscle. For the legs, the center slice was located at the origin of the *popliteus* muscle. FF mapping was performed with a chemical-shift-based (Dixon) 3D gradient-echo sequence (3 echoes, TE =  2.75, 3.95, 5.15 ms, TR =  10 ms, flip angle 3°, resolution 1 x 1 x 5 mm³, 64 slices). A 2D multi-echo spin-echo (MSE) sequence was employed for water T2 mapping [31] (17 echoes, TE =  n x 9.5 ms, TR =  3000 ms, in-plane resolution 1.4 x 1.4 mm², slice thickness 10 mm, 9 slices with 9 mm slices gap). To calculate the transmit field (B_1_^+^), a B_1_^ + ^-mapping sequence (turbo fast low-angle-shot imaging sequence [32]) was acquired covering the same slices as the 2D MSE. For water T1 mapping a magnetic resonance fingerprinting (MRF) sequence was used [33] (TE =  2.1 ms, TR =  10,000 ms, in-plane resolution 1.4 x 1.4 mm², slice thickness 10 mm, 5 slices with 4 mm slice gap).

DTI and ^1^H MRS data were acquired in the right leg using a 15-channel knee RF coil (S1 Fig in S1 Data). For DTI, a stimulated-echo echo-planar imaging DTI sequence (b-value 400 s/mm², 6 diffusion directions, TE =  37 ms, TR =  5,000 ms, in-plane resolution 2 x 2 mm², slice thickness 6 mm, 9 slices with 3 mm slice gap, parallel imaging factor 2, partial Fourier factor 6/8) was used. Spectral adiabatic inversion-recovery (SPAIR) fat suppression was applied to suppress the main fat peak (aliphatic methylene groups at 1.3 ppm) and additional Dixon-based olefinic fat suppression (DOFS) [34] with six readout shifts of 1.1, 3.3, 5.5, 7.8, 10.0, and 12.2 ms was employed. To explore the sensitivity to microstructural changes at longer diffusion times, as suggested in previous works [35, 36], DTI data were acquired at four mixing times of 100, 200, 300, and 400 ms, resulting in diffusion times of 116.3, 216.3, 316.3, and 416.3 ms. Single-voxel ^1^H MRS data were obtained by a point-resolved spectroscopy (PRESS) sequence [25] (TE =  30 ms, TR =  3000 ms). The voxel was positioned at the mid-muscle belly in the right *gastrocnemius medialis* muscle (volume 20 x 20 x 20 mm³). A water-suppressed spectrum (64 averages) and an unsuppressed spectrum (16 averages) were obtained.

The scan time of the whole MRI protocol was approximately 40 minutes.

### MRI data processing

Segmentation was performed using the ITK-SNAP software tool [37] (www.itksnap.org). Muscle regions of interest (ROIs) were manually drawn on the five central first-TE MSE images in 11 muscles of the thighs (*adductor longus* (AL), *adductor magnus* (AM), *biceps femoris long head* (BF), *gracilis* (GRA), *rectus femori*s (RF), *sartorius* (SAR), *semimembranosus* (SM), *semitendinosus* (ST), *vastus intermedius* (VI), *vastus lateralis* (VL) and *vastus medialis* (VM)) and 7 muscles of the legs (*extensor digitorum* (ED), *gastrocnemius lateralis* (GL), *gastrocnemius medialis* (GM), *peroneus longus* (PER), *soleus* (SOL), *tibialis anterior* (TA), *tibialis posterior* (TP)), and interpolated to the Dixon and MRF images for assessment of FF, water T2 and water T1. For DTI parameters, ROIs were drawn on all available slices on the first b =  0 s/mm² image. Additionally, muscle group ROIs were drawn on the out-of-phase Dixon image (five co-registered slices of MSE) to assess the cross-sectional area (CSA) and also FF in muscle groups of thighs (*hamstrings* (HSTR =  BF, SM, ST) and *quadriceps* (QUAD =  RF, VI, VL, VM)) and legs (*anterior compartment* (ANT =  TA, TP, ED), *fibularis* (FIB =  PER), *triceps surae* (TRIC =  GM, GL, SOL).

All further data processing was done in Python using in-house developed software. Fat-water separation of the Dixon data was performed using a three-point Dixon approach with a single T2 * decay and a single-peak fat spectrum. FF maps were calculated as (fat signal)/(water signal +  fat signal)· 100. The contractile CSA (cCSA) was derived as cCSA =  (1–0.01·FF)·CSA. The water T2 values were obtained by tri-exponential fitting, with voxels with water T2 <  15 ms or water T2 >  70 ms, and voxels with B_1_^ +^ values <  80% and >  120% of the prescribed flip angle excluded from further analysis. Water T1 values were obtained by matching signals from a dictionary of fingerprints, and voxels with an FF >  60% were excluded from further analysis. Further details on water T2, FF, and water T1 data processing can be found in previously published works [31,33].

The DTI data for each TM were denoised using an overcomplete local principal component analysis [38], and the estimated noise sigma was used to estimate the signal-to-noise ratio (SNR) at b =  0 s/mm² according to SNR =  mean(DTI signal)/ standard deviation(noise). Subsequently, fat-water decomposition to separate the olefinic fat and the water signal was performed using an adjusted version of the original DOFS algorithm described in [34]. The details are described in S1 Text in S1 Data. The b-values were corrected for the contribution from imaging gradients and non-linear least squares fitting was performed to obtain the diffusion tensor. The tensor eigenvalues (λ_1_, λ_2_, λ_3_), mean diffusivity (MD), and fractional anisotropy (FA) were calculated at each diffusion time as outcome measures. Voxels with an SNR <  15 or olefinic FF >  10% were excluded from further analysis. Moreover, muscles with an overall FF >  50% were excluded from the DTI analysis.

The mean value per muscle averaged over all segmented slices was calculated for each qMRI-based outcome measure. Muscles with <  50 voxels were excluded. Additionally, the average of all muscles, and of all leg muscles (global leg) and all thigh muscles (global thigh) was calculated, weighted by the number of pixels per segmentation. For the comparison of qMRI data with the strength data, the weighted averages per muscle group (QUAD, HSTR, ANT, TRIC) were calculated.

The intramuscular pH values were obtained from the ^1^H MRS data based on the frequency difference between the carnosine (C2H) and the water resonances, as described previously [25]. In muscles with a highly elevated FF (>30%), accurate assessment of intramuscular pH using the carnosine (C2H) resonance was hindered by inadequate signal-to-noise ratio (SNR) and therefore omitted.

### Functional measures

At each visit, the LGMD-R9 cohort of study GNT-015-FKRP performed a comprehensive series of tests. The functional tests were always performed after the MRI to avoid introducing a bias in the qMRI data. First, a forced vital capacity (FVC) test in a seated position was done to assess pulmonary function. The FVC in liter was measured using a spirometer and expressed as a percentage of the expected value for an individual of the same age, height, and race. Second, a series of timed functional assessments were performed, which included 6-minute walk test (6MWT), 10 m walk test (10mWT), time up and go (TUG), and time to climb and descend four stairs (4S climb and descend). The 6MWT was expressed in meters and as the percentage of predicted values [39], and the 10mWT in speed (m/s). Additionally, motor performance was evaluated using the North Star Assessment for Limb-Girdle Type Muscular Dystrophies (NSAD) [40, 41], which consists of 29 items to evaluate the upper and lower limb motor abilities, with a maximum achievable score of 54 points and a higher score indicating higher motor abilities. Finally, isometric muscle strength was measured on the right side for knee flexion and extension and ankle dorsi- and plantarflexion using a Biodex dynamometer and expressed both in absolute values in Newton-meter (Nm) and in percentage of predicted values [42, 43]. All assessments were conducted by trained clinical evaluators and were always performed in the same order. The whole assessment took approximately 2–3 hours and patients could rest between the tests if needed.

### Statistical analysis

Statistical analyses were performed in SPSS (SPSS Statistics 28.01.1.1, IBM Corp., Armonk, NY, USA). The significance level was set at p <  0.05 unless stated otherwise.

The age and BMI distribution between the two groups were compared using a Mann-Whitney U test. qMRI parameters at baseline between the left and right limbs were compared using a Wilcoxon-signed rank test.

For each qMRI parameter, differences between controls and the LGMD-R9 cohort at baseline were assessed using a linear mixed model (LMM) with muscle and group (LGMD-R9/control) as fixed factors and the subject as random factor. The age and BMI were added as covariates. The qMRI parameters FF, cCSA, water T2, and water T1 were assessed, and the DTI indices at diffusion time 116.3 ms were included (MD, FA, λ_1_, λ_2_, λ_3_). Correction for multiple comparisons (9 parameters) was performed and the significance level was set to p <  0.05/9 =  0.0056. Post-hoc tests were performed per muscle. To assess differences in the DTI data between patients and controls at each diffusion time the same LMM was used, with correction for 5 DTI parameters *  4 diffusion times =  20 comparisons (significance level at p <  0.05/20 =  0.0025). pH values between patients and controls were compared using a Mann-Whitney U test. Intramuscular tissue pH was not included in the LMM analysis as data was only obtained in one muscle and fewer data points were available.

In all qMRI parameters with significant changes between the two groups at baseline, a longitudinal analysis was performed to assess changes over time. An LMM was used with muscle and visits as fixed factors and age, BMI, and years since diagnosis as covariates. Visit and muscle were assessed as repeated factors. The same LMM was used to assess changes over time in the functional and strength data (FVC, 6MWT, 10mWT, TUG, 4S climb and descend, NSAD, strength measurements). A Friedman test was used to assess changes in intramuscular tissue pH over time.

The standardized response mean (SRM) for disease progression-sensitive biomarkers FF, cCSA, FVC, 6MWT, 10mWT, TUG, 4S climb and descend, NSAD, and strength measurements was calculated as mean change over time divided by the standard deviation of the change. For FF and cCSA, SRM was calculated per muscle/muscle group and over the global thigh and global leg.

The Spearman correlation coefficients were calculated between FF, water T2, water T1, NSAD, FVC, age, and years since diagnosis, FVC, 6MWT, 10mWT, TUG, 4S climb and descend, and NSAD. The weighted averages over all muscles per participant at baseline from the patients and controls were used. For the correlation of MRI parameters with the strength measurements, the weighted average per muscle group was used, correlating knee extension strength with QUAD, knee flexion with HSTR, ankle dorsiflexion with ANT, and ankle plantarflexion with TRIC. The absolute strength in Nm was correlated with cCSA and the predicted strength in % with FF. The DTI parameters at each diffusion time were correlated with FF, water T1, water T2, age, and years since diagnosis. A correlation coefficient between 0–0.19 was considered as very weak, 0.20–0.39 weak, 0.40–0.59 moderate, 0.60–0.79 strong, and 0.80–1 very strong [44].

To investigate the predictive value of disease activity-sensitive qMRI biomarkers, Spearman correlation analysis between baseline parameters of disease activity (namely, water T2, water T1, pH, DTI parameters) with the change over 2 years in disease progression-sensitive parameters (namely, FF, functional, and strength data), calculated as Δ =  parameter(2-year follow-up) – parameter(baseline), was performed. For ΔFF, the correlation was calculated per muscle and for the global leg and thigh, and for the functional measures only for the global leg and thigh. The correlations with strength were performed on a muscle group level. As pH and DTI were only obtained in the leg, baseline parameters were only correlated with ΔFF, but not with the functional measures.

## Results

### Participant population and data quality

**Fig 1:**
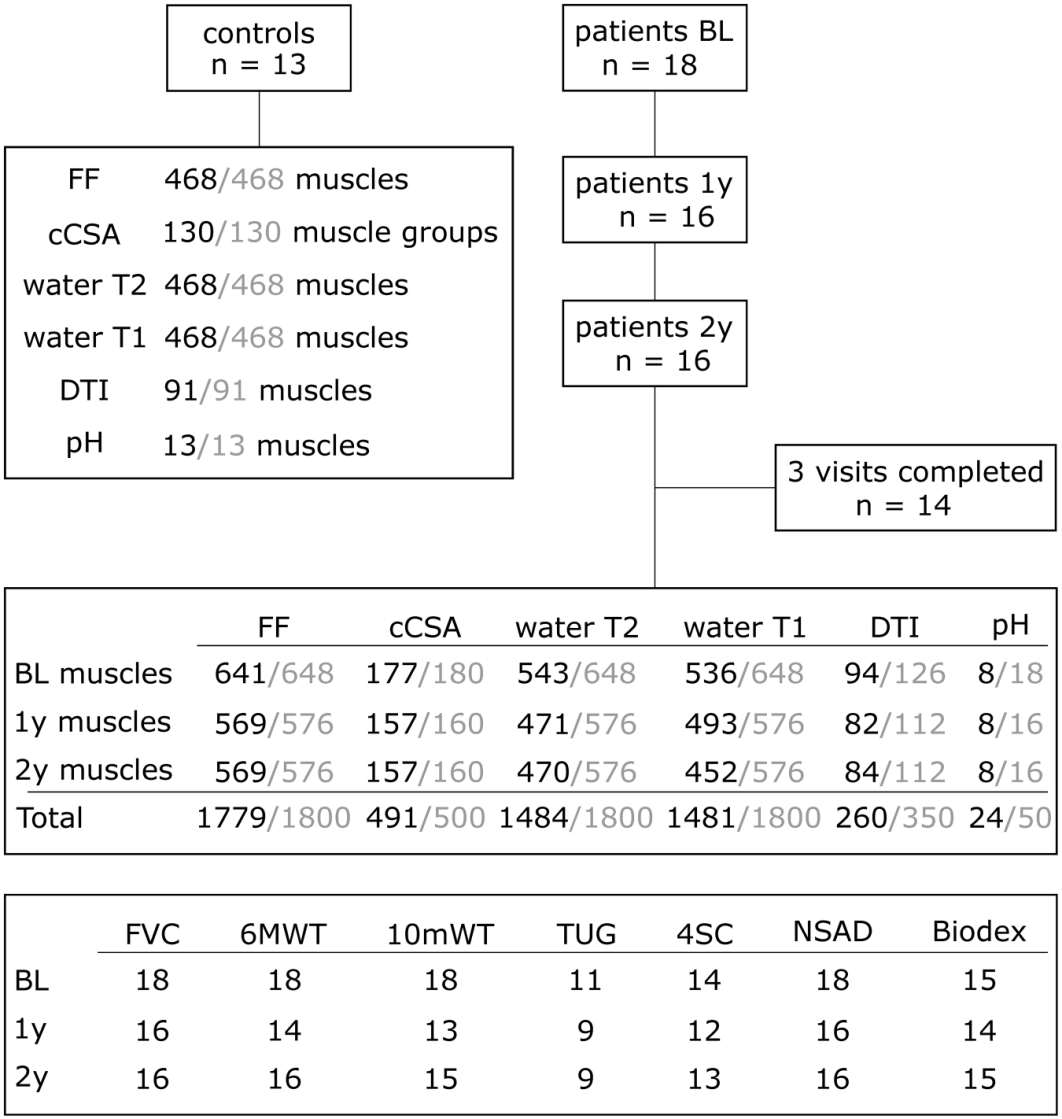
Flow chart of participant inclusion. Flow chart of the participant inclusion and the number of included muscles for all MRI measures and the number of participants for the functional and strength tests. The total number of segmented muscles per visit and MRI measure is given in gray. BL, baseline; cCSA, contractile cross-sectional area; FF, fat fraction; FVC, forced vital capacity; DTI, diffusion tensor imaging; NSAD, North Star Assessment for Limb-Girdle Type Muscular Dystrophies; 6MWT, 6 minute walk test; 10mWT, 10 m walk test; 4SC, 4 stair climb and descend test.

Eighteen individuals with genetically confirmed diagnosis of LGMD-R9 (17f/1m, mean age 38.3 years, range 19–62 years, mean BMI 24.02 kg/m², range 15.24–38.75 kg/m²) and 13 healthy controls (12f/1m, mean age 37.5 years, range 19–67 years, mean BMI 22.44 kg/m², range 18.37–27.68 kg/m²) were included in this analysis. The patients and controls had a similar age distribution and BMI (Mann Whitney U test: p =  0.8/ 0.39 for age and BMI, respectively). All patients completed the baseline scan and at least one follow-up scan (Fig 1). Fourteen patients completed all three visits. There were no baseline differences between the patients with complete follow-up and the patients with one follow-up. The number of years since diagnosis at baseline was 9.4 (range 1.5–17.9). Sixteen patients had the c.826C > A genetic mutation (fifteen homozygous, one heterozygous), and two patients had other mutations (one homozygous, one compound heterozygous). The functional and strength tests could not be completed by all participants at all three visits (Fig 1).

In total, 2268 muscles were segmented [(18 (BL) +  16 (1-year) +  16 (2-year) patients +  13 controls) *  (7 leg +  11 thigh muscles) *  2 (right/left)], whereby 1800 in the patient group and 468 in the controls. No muscles from the control group were excluded. The muscles of the left leg of one patient had to be excluded at all visits due to an implant that caused artifacts (n =  21 muscles, 7 per visit). In the water T2 data, a total of 316 muscles were excluded (21 due to the implant, 295 due to <  50 pixels in the ROI). 319 muscles were excluded from the water T1 data due to the implant (n =  21), corrupted data (n =  180) or FF >  60% (n =  118). In the DTI data, 441 muscles were segmented [(18 (BL) +  16 (1-year) +  16 (2-year) patients +  13 controls) *  7 muscles in the right lower leg]. In total, 90 muscles had to be excluded due to number of pixels <  50 (n =  60) or overall FF >  50% (n =  30). ^1^H MRS data were obtained in 8 patients for all visits. Of the remaining patients, SNR of the carnosine C2H resonance was too low to enable adequate pH determination due to FF being too high.

**Fig 2:**
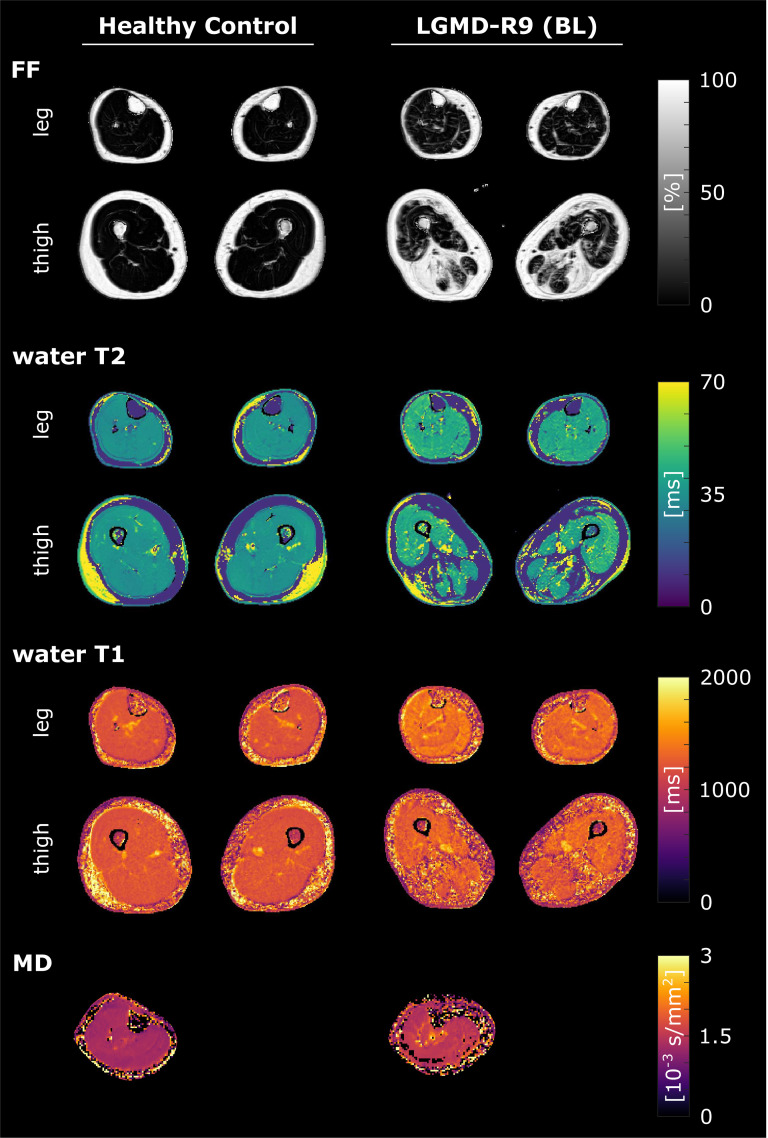
qMRI parameter maps. Parameter maps from the legs and thighs of a healthy control (54y) and a representative individual with LGMD-R9 (62y, 8.6 years since diagnosis). In the patient elevated FF, water T2, and water T1 are visible in both, legs and thighs. FF, fat fraction; BL, baseline; MD, mean diffusivity.

Example parameter maps of a healthy control and a patient are shown in Fig 2.

Significant differences between the left and right leg were found in FF and water T1 in both patients and controls (FF: p <  0.001/ < 0.001, water T1: p <  0.001/ < 0.001) and in cCSA in the patient group (p =  0.032), but not in the controls (p =  0.528). However, the absolute differences were small (FF <  2% in patients/ < 0.5% in controls, water T1 <  10 ms in both groups). No differences were found for water T2 in patients or controls (p =  0.530/0.563). Nevertheless, for all further analyses, the data from the right and left limbs were averaged.

### qMRI differences between controls and LGMD-R9 at baseline

Significant differences between the patient and control group were found for FF (p <  0.001), water T2 (p =  0.002), water T1 (p =  0.003), and cCSA (p =  0.001), but not for any DTI parameter at diffusion time 116.3 ms. P-values for MD, FA, λ_1_, λ_2_, and λ_3_ were 0.232, 0.774, 0.296, 0.098, and 0.688, respectively. Intramuscular tissue pH was higher in patients than in controls (p =  0.016).

**Fig 3:**
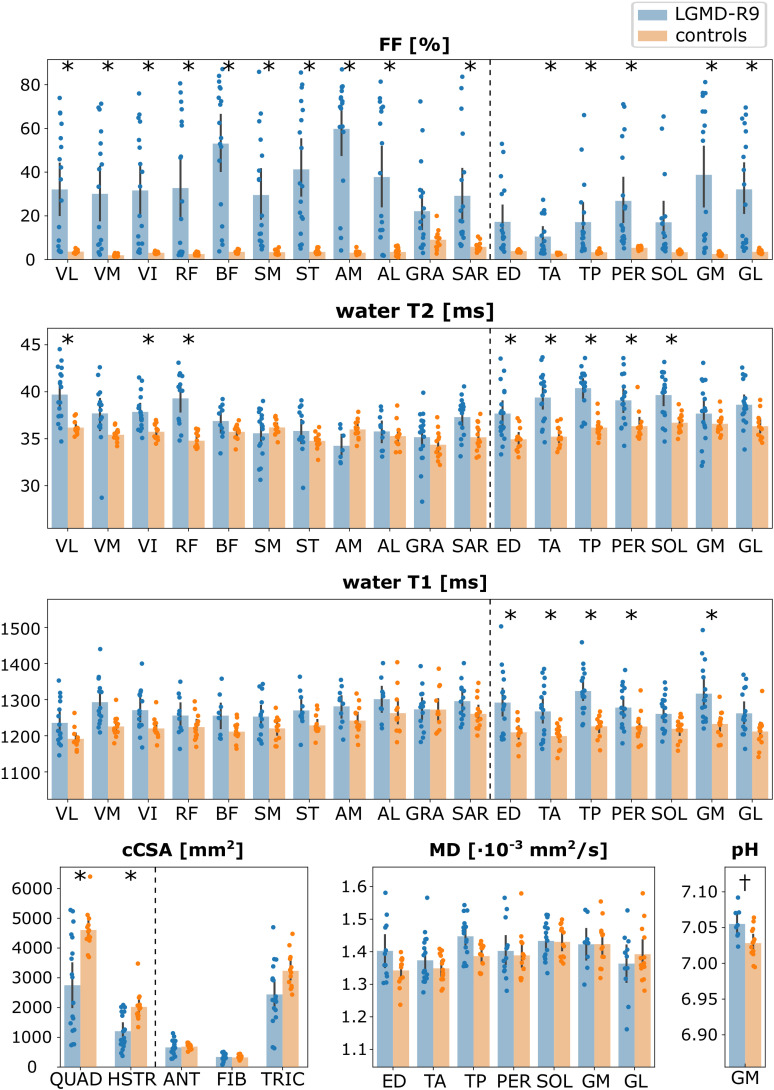
Bar plots of qMRI parameters. Bar plots comparing the LGMD-R9 at baseline (blue) and the controls (orange), showing the mean ± standard deviation, for FF, water T2, water T1, cCSA, MD at diffusion time 116.3 ms, and pH, for all muscles. The individual data points are overlaid as dots. Muscles from the thighs and legs are separated by the dotted line. cCSA, contractile cross-sectional area; FF, fat fraction; MD, mean diffusivity. Muscles in the order of appearance: VL, *vastus lateralis*; VM, *vastus medialis*, VI, *vastus intermedius*; RF, *rectus femoris*; BF, *biceps femoris long head*; SM, *semimembranosus*; ST, *semitendinosus*; AM, *adductor magnus*; AL, *adductor longus*; GRA, *gracilis*; SAR, *sartorius*; ED, *extensor digitorium*; TA, *tibialis anterior*; TP, *tibialis posterior*; PER, *peroneus longus*; SOL, *soleus*; GM, *gastrocnemius medialis*; GL, *gastrocnemius lateralis*; QUAD, *quadriceps*; HSTR, *hamstrings*; ANT, *anterior compartment*; FIB, *fibularis*; TRIC, *triceps surae*. *p<0.0056 (= 0.05/9). †p<0.05.

Post-hoc analysis per muscle demonstrated significant differences in FF between the two groups in all muscles except for GRA, ED, and SOL (p =  0.023, 0.010, and 0.006, respectively). The highest FF in the thighs was found in the AM, followed by the BF muscle, and in the legs in the GM, followed by the GL (Fig 3, Table 1). The VM in the thighs and the TA in the legs presented the lowest FF. A large spread in FF values between individual patients was observed (Fig 3). For cCSA, significant differences with the control group were found in the muscle groups of the thighs (p-values for HSTR and QUAD were 0.001 and 0.004), but not the legs (p-values for ANT, FIB, and TRIC were 0.650, 0.567, and 0.012).

**Table 1:** FF per muscle at each visit. Mean FF (standard deviation, SD) per muscle at each visit and mean change (SD) in FF (ΔFF) after 1-year (1y) and 2-year (2y) follow-up in patients. Only data of the 14 patients who completed all 3 visits are shown here. The mean value of all muscles and the global thigh and leg were added at the bottom. The most and least affected muscles of the thigh and leg and the muscles with the most and least increase over 2 years are highlighted in red and blue, respectively. BL, baseline; FF, fat fraction.

muscle	Controls	LGMD-R9 BL	LGMD-R9 1y	LGMD-R9 2y	ΔFF(1y – BL)	ΔFF(2y – BL)
**VL**	3.6 (0.8)	29.6 (26.6)	30.4 (26.9)	31.7 (26.6)	0.7 (2.9)	2.1 (5.4)
**VM**	2.0 (0.6)	27.0 (26.1)	27.4 (26.4)	28.8 (26.1)	0.4 (2.2)	1.9 (4.0)
**VI**	3.1 (0.5)	28.6 (25.3)	29.7 (26.1)	30.1 (24.7)	1.1 (1.9)	1.5 (4.1)
**RF**	2.5 (0.6)	33.1 (32.0)	34.7 (33.1)	35.1 (32.9)	1.5 (2.3)	2.0(4.5)
**BF**	3.5 (0.7)	51.1 (32.1)	52.6 (32.1)	53.4 (31.5)	1.5 (3.8)	2.2 (6.0)
**SM**	3.4 (1.1)	31.9 (27.8)	33.5 (28.0)	34.2(27.9)	1.6 (3.1)	2.3 (5.1)
**ST**	3.5 (1.1)	42.1 (31.8)	43.4 (31.5)	44.2 (30.8)	1.2 (2.5)	2.1 (3.9)
**AM**	3.1 (0.9)	58.0 (27.3)	59.2 (27.2)	60.3 (28.1)	1.2 (3.0)	2.3 (4.8)
**AL**	3.5 (1.7)	38.3 (32.2)	40.4 (32.1)	41.5 (31.1)	2.1 (3.4)	3.2 (5.6)
**GRA**	9.1 (4.5)	23.3 (21.8)	24.4 (23.2)	25.8 (21.8)	1.1 (3.8)	2.5 (5.5)
**SAR**	5.8 (2.2)	31.9 (28.3)	33.9 (28.8)	34.7 (28.1)	2.0 (3.0)	2.8 (5.4)
**ED**	3.9 (0.5)	14.3 (14.3)	16.0 (15.9)	14.9 (14.2)	1.7 (2.4)	0.6 (2.5)
**TA**	2.7 (0.3)	9.3 (8.2)	10.7 (10.8)	10.5 (9.7)	1.4 (3.2)	1.1 (3.4)
**TP**	3.4 (0.7)	17.3 (19.2)	18.1 (19.1)	18.2 (18.0)	0.8 (3.6)	0.9 (4.6)
**PER**	5.4 (0.7)	24.1 (23.7)	25.2 (23.8)	25.1 (23.1)	1.2 (3.4)	1.1 (3.9)
**SOL**	3.4 (0.6)	17.8 (21.2)	18.7 (21.5)	18.9 (21.3)	0.9 (3.06)	1.1 (4.1)
**GM**	2.6 (0.6)	35.2 (33.9)	36.0 (34.1)	36.6 (33.7)	0.8 (2.0)	1.4 (3.4)
**GL**	3.6 (0.8)	29.6 (26.1)	31.1 (27.7)	32.0 (28.1)	1.5 (4.1)	2.3 (3.8)
**Mean all**	3.3 (0.6)	30.6 (24.2)	31.5 (24.4)	32.3 (24.1)	0.9 (1.3)	1.7 (3.3)
**Global thigh**	3.3 (0.7)	35.3 (27.1)	36.5 (27.2)	37.3 (26.6)	1.2 (1.5)	2.1 (3.7)
**Global leg**	3.3 (0.5)	22.8 (21.8)	23.9 (22.8)	24.3 (22.4)	1.1 (2.8)	1.5 (3.7)

For water T2, differences were found in the anterior compartment of the thighs (RF, VI, VL) and legs (ED, PER, SOL, TA, TP). Similar to FF, the spread of water T2 values was larger in patients than in controls. Water T1 showed significant differences in all leg muscles except SOL and GL, but not in any thigh muscle.

**Fig 4:**
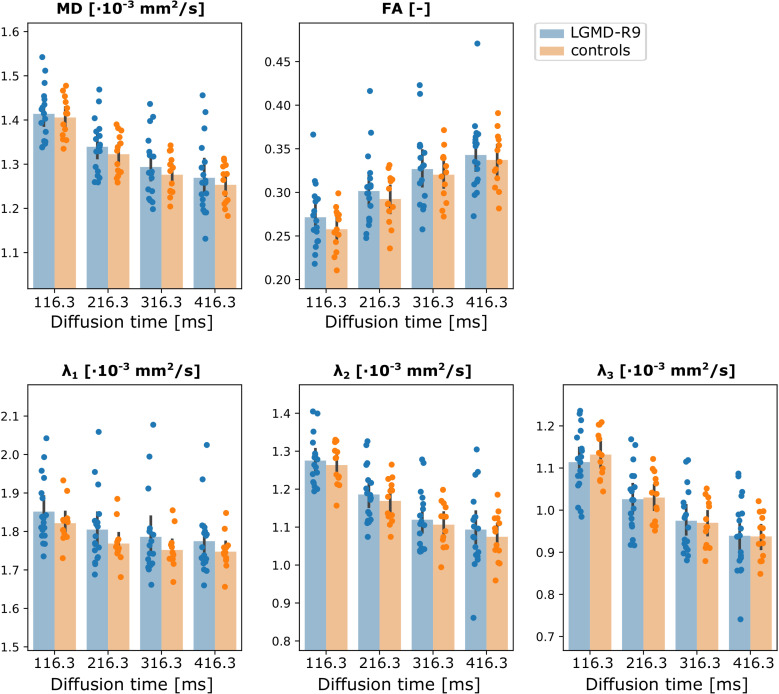
Bar plots of DTI parameters. Bar plots comparing the DTI parameters between LGMD-R9 at baseline (blue) and controls (orange) in the global leg at all diffusion times. DTI, diffusion tensor imaging; FA, fractional anisotropy; λ_1_- λ_3_, diffusion tensor eigenvalues; MD, mean diffusivity.

Comparing the DTI parameters at each diffusion time, no significant differences were found in any parameter at any diffusion time (Fig 4). Nevertheless, a larger spread in DTI indices was found in the patients, which was especially pronounced in MD and λ_1_ (Fig 4).

### Longitudinal qMRI analysis in patients

In the 14 patients who completed all three visits, an overall increase in FF at 1-year and 2-year follow-up was observed in all muscles, with a larger increase in the thighs (Table 1). Yet, the changes in FF did not reach significance (p-value =  0.566). Similarly, no significant changes over time were found for cCSA (p =  0.946), water T2 (p = 0.234), water T1 (p =  0.467), and pH (p =  0.882).

**Fig 5:**
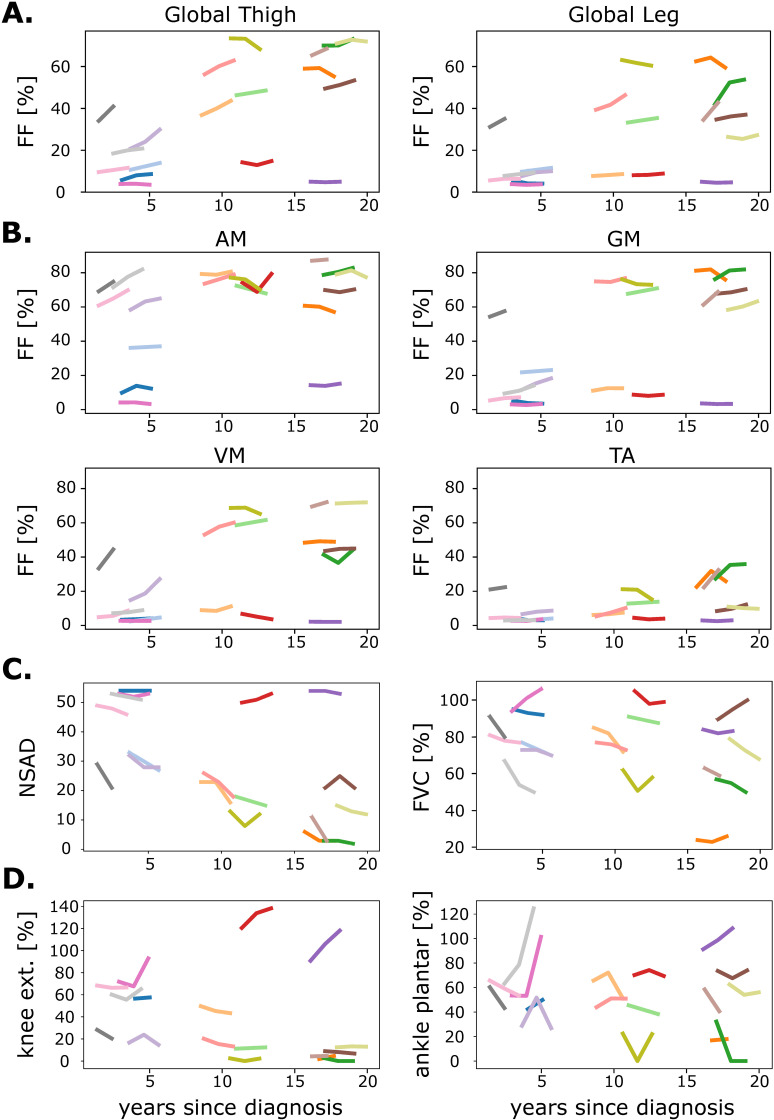
Time course of qMRI, functional, and strength measurs. Line plots showing the time course over the years since diagnosis. Each line represents an individual with LGMD-R9 and the same colors show the same participant. **A.** FF in the global thigh and global leg. **B.** FF in the most and least fat-replaced thigh and leg muscles (AM, VM, GM, TA). **C.** Functional measures NSAD and FVC. **D.** Strength tests for knee extension and ankle plantarflexion. The predicted strength is shown. AM, *adductor magnus*; VM, *vastus medialis*; GM, *gastrocnemius medialis*; TA, *tibialis anterior*; FF, fat fraction; FVC, forced vital capacity; NSAD, North Star Assessment for Limb-Girdle Type Muscular Dystrophies.

Examining individual patients revealed a notable variation in FF trajectories over time, as shown in Fig 5. While the increase in FF was mild to moderate in most patients, some patients remained stable over the 2-year observation period (Fig 5).

The SRM values for FF and cCSA did not exceed 0.8 (S1 Table in S1 Data).

### Correlations of qMRI outcome measures

FF was very strongly correlated with years since diagnosis. A moderate to strong correlation with FF was found for water T2, water T1, and pH. Water T1 and water T2 were very strongly correlated. Detailed results from this correlation analysis can be found in S2 Table in S1 Data.

For the DTI parameters, a weak to moderate correlation with FF was found in some parameters for all diffusion times. A weak correlation with water T2 was observed in the diffusivity at diffusion times >  116.3 ms, but not for FA. The detailed parameters can be found in S3 Table in S1 Data.

### Predictive value of disease activity-sensitive qMRI parameters

**Fig 6:**
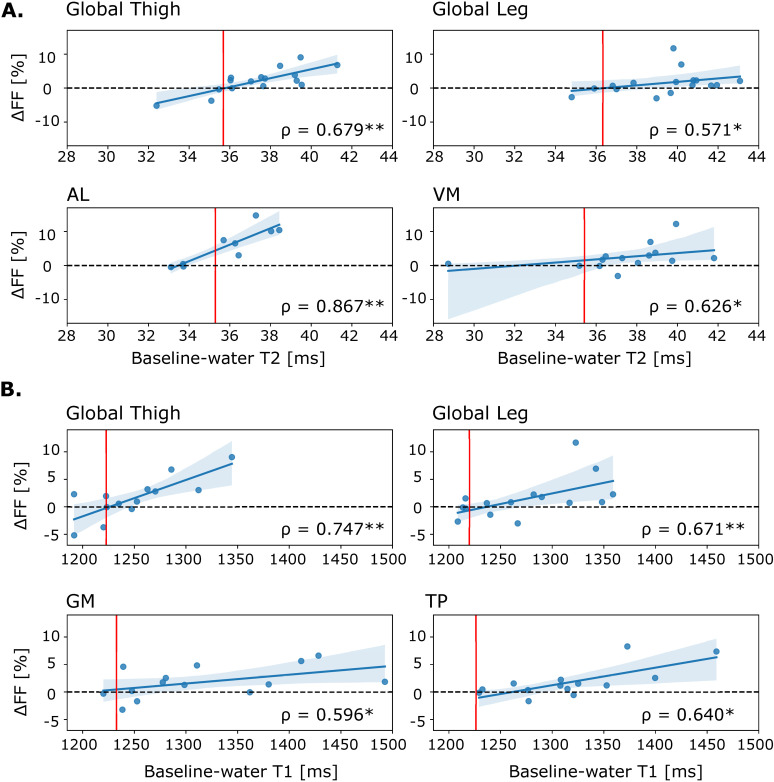
Predictive value of disease activity-sensitive qMRI parameters. Regression plots of ΔFF with **A.** Baseline water T2 and **B.** Baseline water T1 values. The dots represent individuals with LGMD-R9 and the red line indicates the mean value in the controls. AL, *adductor longus*; GM, *gastrocnemius medialis*; TP, *tibialis posterior*; VM, *vastus medialis*. *p < 0.05, **p < 0.01.

A strong significant correlation between baseline water T2 and baseline water T1 with ΔFF was found in the global leg and global thigh (Table 2). No correlation between baseline pH or any DTI parameter at baseline with ΔFF was found (S4 Table in S1 Data). At individual muscle level, strong to very strong correlations between baseline water T2 with ΔFF were found for AL, GRA, RF, SM, ST, VL, and VM. Baseline water T1 was moderately to strongly correlated with ΔFF in the VL, GM, and TP muscles (Fig 6, S5 Table in S1 Data).

**Table 2:** Correlation of baseline water T2 and water T1 with disease progression.

		Baseline water T2		Baseline water T1	
		Leg	Thigh	Leg	Thigh
**ΔFF**		0.571^*^	**0.679** ^**^	**0.671** ^**^	**0.747** ^**^
**ΔFVC**		-0.219	-0.208	-0.224	-0.077
**Δ6MWT**		-0.406	-0.209	-0.279	-0.291
**Δ10mWT**		-0.341	-0.239	-0.207	0.185
**ΔTUG**		0.350	**0.600**	0.550	**0.683** ^*^
**Δ4S climb**		0.522	0.599^*^	0.406	0.067
**Δ4S descend**		0.297	0.154	0.035	-0.188
**ΔNSAD**		-0.250	-0.389	-0.466	-0.236
**ΔKnee flexion**	**HSTR**	0.105		-0.300	
**ΔKnee extension**	**QUAD**	-0.544		-0.479	
**ΔAnkle dorsi flexion**	**ANT**	-0.082		-0.301	
**ΔAnkle plantar flexion**	**TRIC**	-0.418		-0.462	

Spearman correlation coefficient between baseline qMRI parameters of disease activity with the change in FF, functional, and strength measures over 2 years (Δ) for the global leg and thigh. The predicted strength was used for the correlation. Strong correlations are marked in bold.

* p < 0.05,

** p < 0.01.

### Functional and strength data and correlations with qMRI

The functional data suggests a downward trend in several patients, as illustrated for NSAD and FVC in Fig 5. Yet, none of the changes in any functional test reached significance (p-values: FVC =  0.237, 6MWT =  0.575, predicted 6MWT =  0.752, 10mWT =  0.057, TUG =  0.745, 4S climb =  0.651, 4S descend =  0.617, and NSAD =  0.592, respectively). The mean data per visit and the change over 1 and 2 years for all functional measures are given in Table 3. Interestingly, an increase in strength was found for all strength measurements from baseline to follow-up (Table 3), which reached significance for knee flexion (p =  0.015) and ankle dorsiflexion (p =  0.025). On a per-subject basis, the functional and strength measures showed a heterogeneous picture, similar to FF (Fig 5). It should be noted that the increase in strength was found in patients with low FF and high baseline strength (Fig 5 D). The SRM for the functional and strength measurements was <  0.8, except for the 4S climb (SRM =  0.87) (S1 Table in S1 Data).

**Table 3:** Functional and strength parameters.

	N	Baseline	1y	2y	Δ(1y – BL)	Δ(2y – BL)
**FVC [%pred]**	14	76.6 (20.1)	73.8 (21.8)	73.2 (22.4)	-2.8 (5.2)	-3.5 (7.7)
**6MWT [m]**	13	370.2 (178.9)	366.4 (185.4)	358.9 (191.6)	-3.8 (29.3)	-11.3 (20.6)
**6MWT [%pred]**	13	60.4 (29.1)	60.1 (30.3)	58.6 (30.8)	-0.3 (4.7)	-1.8 (3.2)
**10mWT [m/s]**	11	1.5 (0.6)	1.8 (0.8)	1.8 (0.9)	0.3 (0.4)	0.3 (0.5)
**TUG [s]**	8	8.0 (5.1)	8.0 (3.9)	9.0 (6.6)	0.0 (1.3)	1.0 (1.5)
**4S climb[s]**	11	5.7 (4.8)	6.9 (6.5)	7.1 (6.1)	1.1 (3.3)	1.4 (1.6)
**4S descend [s]**	11	4.0 (3.5)	3.8 (2.6)	4.3 (3.4)	-0.2 (1.1)	0.3 (0.7)
**NSAD [-]**	14	32.3 (10.4)	31.2 (20.0)	30.1 (20.5)	-1.1 (2.2)	-2.1 (2.8)
**Knee flexion [Nm]**	9	34.6 (22.9)	32.4 (19.8)	38.3 (26.0)	-2.2 (5.4)	3.7 (7.1)
**Knee flexion [%pred]**	10	58.6 (33.2)	56.3 (29.2)	67.0 (41.4)	-2.3 (10.0)	8.3 (14.3)
**Knee extension [Nm]**	10	59.8 (43.4)	60.2 (45.7)	64.9 (52.9)	0.5 (7.8)	5.1 (11.5)
**Knee extension [%pred]**	10	51.9 (37.4)	53.5 (41.7)	57.2 (47.3)	1.6 (7.2)	5.2 (11.6)
**Ankle plantarflexion [Nm]**	10	63.6 (17.3)	67.8 (16.5)	74.1 (35.4)	4.3 (10.0)	10.5 (26.8)
**Ankle plantarflexion [%pred]**	10	61.9 (17.0)	66.1 (15.3)	71.7 (30.7)	4.2 (9.5)	9.8 (24.2)
**Ankle dorsiflexion [Nm]**	10	19.8 (9.2)	22.9 (10.5)	22.4 (8.8)	3.1 (4.3)	2.5 (3.2)
**Ankle dorsiflexion [%pred]**	10	80.1 (25.7)	93.6 (30.2)	92.1 (28.1)	13.5 (17.6)	12.0 (15.0)

Functional parameters at all visits and their change over time (Δ) after 1-year (1y) and 2-years (2y) in the individuals with LGMD-R9 who completed all 3 visits. The mean (standard deviation) is shown. BL: baseline; N: number of participants with 3 visits for each test; %pred: % of predicted value.

Regarding the correlation between qMRI measures and functional measures, 6MWT, 10mWT, and NSAD were very strongly negatively correlated with FF, and a strong negative correlation between FF and FVC was found. The functional measures TUG and 4S climb and descend were strongly correlated with FF. Moreover, 4S climb and descend and TUG showed a strong to very strong correlation with water T1 and water T2 (S2 Table in S1 Data).

Similarly, the strength measurements were all very strongly correlated with cCSA and the predicted strength was moderately to very strongly negatively correlated with FF (S6 Table in S1 Data). Moreover, the knee extension and flexion were moderately to strongly negatively correlated with the years since diagnosis.

Regarding the predictive function of water T2 and water T1, a strong correlation between baseline water T1 in the thighs and ΔTUG and a moderate correlation between baseline water T2 in the thighs and Δ4S climb were found. For baseline water T2 in the thigh, a strong correlation with ΔTUG was found; however, it did not reach significance (p = 0.088). No correlation with the other functional measures was observed (Table 2). Baseline water T2 and baseline water T1 were not correlated with any strength measure at muscle group level (Table 2).

## Discussion

In this sub-cohort of 18 individuals with LGMD-R9 of the GNT-015-FKRP natural history study, we found elevated muscle FF, water T2, water T1, and intramuscular pH values in lower limb muscles compared to control participants. The baseline water T2 and water T1 were strongly correlated with the increase in FF and TUG over a 2-year follow-up period, and baseline water T2 in the thighs correlated with the increased time of 4S climb, indicating that water T2 and water T1 have a predictive value for disease progression. No changes over time in qMRI parameters and functional tests were observed across the two-year study in this cohort.

The FF in the LGMD-R9 cohort was 3–18 times higher than in the controls, with an overall higher FF in the thighs than in the legs. The highest FFs were found in the posterior muscles of the thighs and legs. This is in line with previous LGMD-R9 studies that reported the most severe involvement in the posterior compartment, with the most affected muscles being the BF and adductors [20,21,45,46]. A relative sparing of the anterior compartment, and especially VL and TA, has been reported [46], which is also reflected by our results.

Besides FF, elevated water T2, water T1, and intramuscular tissue pH were found in this study. While this has not been studied in LGMD-R9 before, elevated water T2 has been reported in other LGMDs like LGMD-R1 (calpainopathy) [19,24], LGMD-R2 (dysferlinopathy) [16], and other muscular dystrophies [47], and has been associated with active muscle damage. Interestingly, Arrigoni et al. reported reduced water T2 in calpainopathy and dysferlinopathy patients (LGMD-R1/R2, formally 2A/2B) [48], and Forsting et al. in the posterior muscles in LGMD-R1 [19]. However, in both studies, those muscles had FF close to or above 60% and a decrease in highly fat-replaced muscles has been associated with susceptibility differences of fat and water [49]. More recently, it has been shown that water T1 is sensitive to disease activity and correlates with water T2 [23,50]. We also found a very strong correlation between water T2 and water T1. An advantage of water T1 over water T2 is the faster acquisition time with an MRF sequence, which could in the future be used to accelerate the scan protocol. Furthermore, water T1 can be combined with gadolinium-enhanced imaging to estimate the extracellular volume (ECV). Recently, an increased ECV was found in Becker muscular dystrophy, but no abnormal water T2 or water T1 [51], thus ECV could provide more insights into the disease mechanism. The elevated intracellular pH found in LGMD-R9 patients with low FF further implies increased disease activity and suggests altered ionic homeostasis within the muscle cells indicative of changes at the level of the sarcolemma.

Interestingly, no changes between patients and controls were observed in the DTI parameters at any diffusion time. DTI had not been performed in LGMD-R9 before. However, previous studies in LGMD-R1/D4 and LGMD-R2 (calpainopathy/dysferlinopathy) reported elevated FA and decreased diffusivity in muscles with FF <  70% [48] and in high-risk muscles before muscle-fat replacement (FF <  8%) [24]. Contrary, no changes in DTI parameters at diffusion times up to 300 ms were found in Becker muscular dystrophy [52, 53], which has a similar phenotype as LGMD-R9 [54]. The discrepancy might be explained by the different disease mechanisms of LGMD-R1, -R2, and –R9. For example, dysferlinopathy shows defects in the membrane repair system [55], which could result in leaky membranes and thus altered diffusion parameters. However, in this case, an increase in diffusion would be expected. As no additional olefinic fat suppression was applied in the previous studies, and the cut-off FF was 70%, the reported DTI indices might have been partly influenced by residual fat, as fat can result in an underestimation of diffusivity and an overestimation of FA [56, 57]. In calpainopathy, variations in muscle fiber sizes have been reported [7], which might alter tissue diffusion. While this has also been reported for LGMD-R9 [58], calpainopathy typically presents with a more severe phenotype, thus changes in tissue diffusion might be more pronounced and could be below the detection limit for LGMD-R9. Nevertheless, we found a greater spread in diffusion parameters in patients than in controls, especially in MD and λ_1_, which could indicate alterations in diffusion in some. More advanced diffusion models like the random permeable barrier model might reveal minor changes in microstructure [59, 60] but were beyond the scope of this article. Moreover, using more diffusion directions could improve the stability of the tensor estimates and allow the detection of smaller changes. In this work, 6 diffusion directions were chosen to keep the acquisition time with the multiple readout shifts for DOFS and multiple diffusion times in an acceptable range.

Despite the improved olefinic fat suppression applied in this study, a weak to moderate but significant correlation with FF was found for some diffusion parameters. This could potentially obscure subtle changes in the DTI parameters. However, without olefinic fat suppression, a moderate to very strong correlation of all diffusion parameters with FF has recently been reported in facioscapulohumeral muscular dystrophy (FSHD) [61]. Therefore, we conclude that Dixon-based olefinic fat suppression improves the estimation of DTI parameters in individuals with fat infiltration.

In this longitudinal analysis, we found a yearly increase in FF of around 1.1% in the legs and 1.2% in the thighs. This is in agreement with previous findings [13]. Yet, the changes in our study did not reach significance, most likely due to the smaller number of participants compared to previous reports. For example, Willis et al. found significant changes in some muscles after 1 year, however, their study included 38 LGMD-R9 patients [13]. In a study over 6 years, a significant increase in all observed muscles was found, ranging from 1–11% [12]. Also, the cohort in our study was very heterogeneous, with some patients presenting with high FF at baseline already, while others were in an earlier stage of the disease. Given the heterogeneous and slow-progressing nature of LGMD-R9, longer follow-up periods and/or a more homogeneous population might be necessary to detect significant changes in a small cohort. The majority of patients included in this study had the common homozygous mutation (c.826C > A), associated with a known milder phenotype [1], which might also explain the subtle progression over 2 years. No changes over time were found for water T2, water T1, and intramuscular pH. As these are markers of disease activity, they are time-point specific and no progressive increase or decrease over time was expected.

Unlike pH and DTI parameters, we could show that water T2 and water T1 were correlated with ΔFF in the global leg and thigh, and in some individual muscles. This indicates that both water T2 and water T1 have a predictive value for disease progression. The predictive value of water T2 on change in FF has been reported previously in dysferlinopathy, inclusion body myositis, late-onset Pompe disease, and GNE myopathy [16,62–65]. Furthermore, in dysferlinopathy and inclusion body myositis, baseline water T2 correlated with the decline of functional outcome measures and increased FF over 1 and 3 years [63, 64]. We found correlations with ΔFF, and in the thighs, we found correlations of baseline water T1 and water T2 with ΔTUG and Δ4S climb, but not in any other functional or strength measure. It has been shown that contractile properties are close to normal in individuals with LGMD-R9, resulting in an intact cCSA-strength relationship. This is in contrast to Becker muscular dystrophy (with a phenotype closely resembling LGMD R9) where this relationship was perturbed [66]. In dysferlinopathy the cCSA-strength relationship was not investigated but the disease is known to have an impaired membrane repair mechanism [55], which might result in membrane leakage and exercise intolerance and could negatively impact contractile properties, explaining a faster decline of function. The predictive value of water T1 has not been reported before. Interestingly, while in the global leg and thigh both baseline water T2 and water T1 were correlated with ΔFF, differences were found on an individual muscle level. The predictive value of water T2 and water T1 could aid in enabling more tailored care by allowing patient-specific predictions of disease progression at an early stage, or in selecting suitable patients and muscles for interventions. In this study, follow-up data was available for the patient group only. Previous work in healthy cohorts demonstrated excellent reproducibility and repeatability as well as temporal stability over 1.5 years for water T2 and FF [67, 68]. Therefore, we believe that the correlations between baseline water T1 and water T2 with ΔFF found in our study hold clinical value to predict disease progression. However, including follow-up data of the control group in future studies might strengthen the results.

Similar to the qMRI data, no decline in function or strength was observed in our data. The increase we found in some strength measures is most likely attributed to measurement variations. All functional and strength measures correlated strongly to very strongly with FF, which is in line with previous findings [12,20].

This study had some limitations. First, the LGMD-R9 cohort was relatively small and follow-up data were only obtained over 2 years. Given the heterogeneity in the patient group with the number of years since diagnosis ranging from 1.5–18, longer follow-up periods or a more homogeneous patient population are desirable. Furthermore, our study population consisted of 17 females and only 1 male patient, which is not representative of its occurrence in the general population. Second, most patients had already high FF at the start of the study, with an average FF of all muscles of 30%. Those patients were not at an early stage of the disease anymore. Disease activity and microstructural changes are expected before muscle is replaced by fat. The heterogeneity might have limited our ability to detect early changes, especially in DTI. Third, we found small, but significant differences between the left and right leg in FF and water T1 in this study. This is unexpected, as LGMD-R9 is not known to be an asymmetric disease. Although significant, the differences were minor, which does not justify to conclude an asymmetry and should thus not be overemphasized. A possible explanation are B_1_^ +^ inhomogeneities, which can influence image quality and qMRI parameter estimation in the extremities [69,70]. This hypothesis is supported by the fact that these differences were also found in the control group and no left-right differences in water T2 were observed, where only voxels with minimal B_1_^ +^ variations were included. Fourth, DTI data were only obtained in the right lower leg, and ^1^H MRS only in the gastrocnemius muscle. This choice was made to ensure maximal SNR and to study muscles with low FF to identify possible changes before muscle-fat replacement, as the lower legs are typically less affected than the thighs in LGMD-R9. However, given that our patient group presented with an average FF of 22% in the legs and 35% in the *gastrocnemius medialis* muscle at baseline, which is beyond the normal range, future studies might focus on early-stage patients for DTI and intracellular tissue pH measurements. Fifth, the number of years since diagnosis used in this study is not necessarily identical to the number of years since symptom onset. For a better understanding of the time development of LGMD-R9 the number of years since symptom onset would be preferable. However, this data was not available for this study. Sixth, our control group was smaller than our patient group. However, given the excellent reproducibility and small coefficient of variation expected in a healthy cohort [67,68], and the similar age and BMI distribution between our control and patient groups, we consider the smaller number of controls acceptable.

In conclusion, individuals with LGMD-R9 presented increased FF, water T2, water T1, and pH values, but no change in DTI parameters in the lower leg and thigh muscles as compared to healthy controls. Baseline water T2 and water T1 correlated with ΔFF in some muscles over a 2-year follow-up, as well as with the increased times in the functional measures ΔTUG and Δ4S climb, suggesting that water T2 and water T1 are predictive of disease progression. These findings may be valuable for identifying biomarkers and outcome measures in clinical trials or for selecting eligible LGMD-R9 patients in treatment studies.

## Supporting information

S1 Data Containing S1 Fig, S1 Text, S1-S6 Tables .
(PDF)
