# Supplementary material for: Multi-parametric quantitative MRI of the lower limb muscles in a longitudinal study of limb-girdle muscular dystrophy R9
Source: PLoS One. 2025 Apr 28;20(4):e0321463. doi: 10.1371/journal.pone.0321463 (PMC12036925; doi:10.1371/journal.pone.0321463)
Supplement: S1 Data — . (PDF) [file pone.0321463.s001.pdf]

Supporting Information to "Multi-parametric quantitative MRI of the lower limb muscles in a longitudinal study of limb-girdle muscular dystrophy R9"

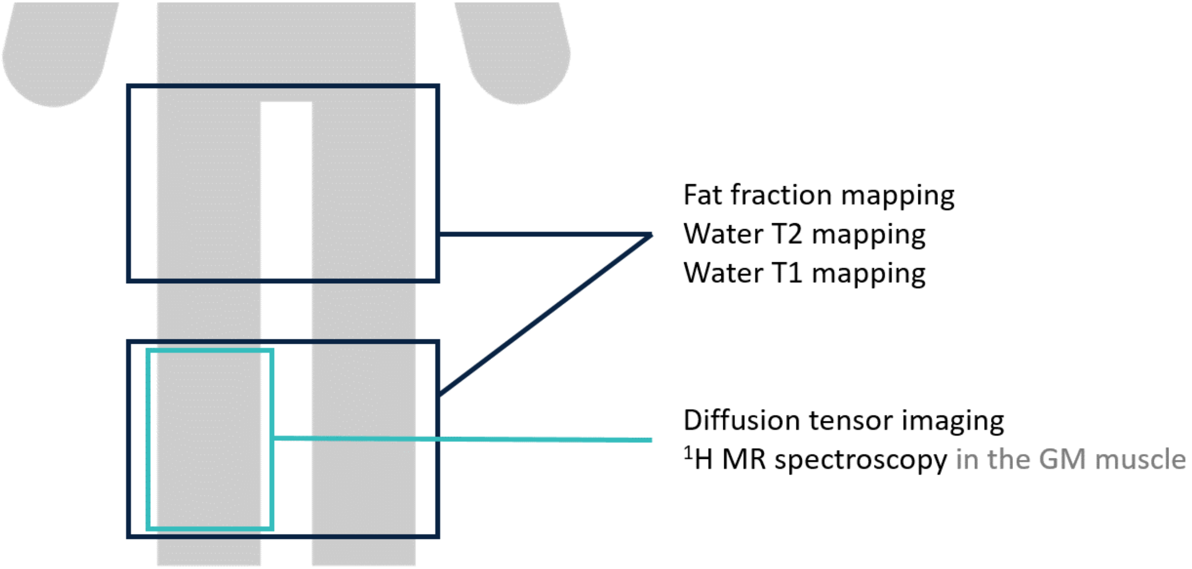

S1 Fig: **Schematic overview of the MRI acquisition**. Fat fraction, water T2, and water T1 mapping were performed in both thighs and both legs. Diffusion tensor imaging was performed in the right lower leg and ^{1}H MRS in the right *gastrocnemius medialis* (GM) muscle.

S1 Text: Detailed DTI data processing

The following steps were performed for the DTI data of each diffusion time.

1. Denoising using an overcomplete local principal component analysis.
2. Registration of the DTI data to the first b = 0 s/mm² volume at diffusion time 116.3 ms using a rigid registration.
3. T2* maps were estimated from the shortest diffusion time data (116.3 ms).
4. DOFS fat-water separation using a dual-echo approach on the six readouts as illustrated below. This resulted in three water maps per b-value, diffusion direction, and diffusion time. T2* correction was applied using the T2* maps calculated in step (3).

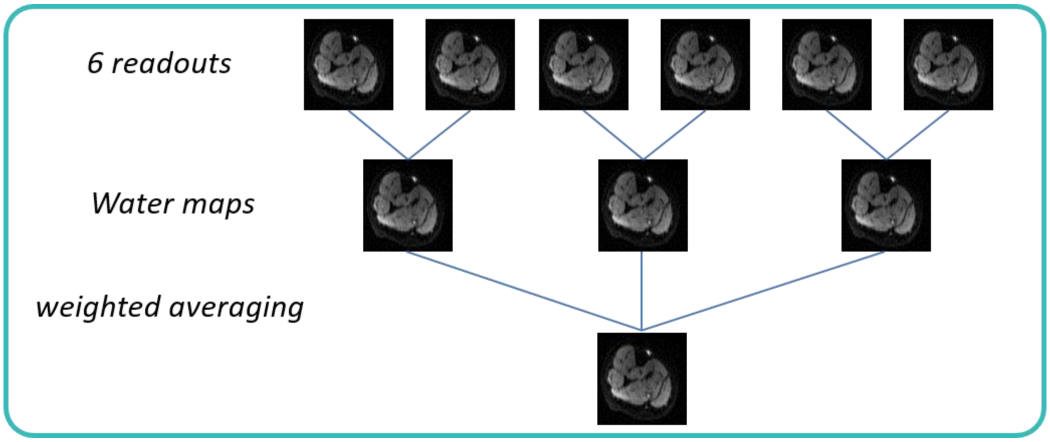

5. Weighted averaging of the water maps for each b-value, diffusion direction, and diffusion time was performed to reduce artifacts arising from muscle twitches. The Gaussian-filtered normalized water maps were used as weights, resulting in higher weights for a higher signal, assuming that muscle twitches cause signal dropouts.
6. Correction of the b-values for contribution from imaging gradients. The corrected b-values per diffusion time and for all gradient directions are given below:

nominal b-value (s/mm²)	0	400	400	400	400	400	400
Diffusion time 116.3 ms	26.66	387.61	387.61	387.61	412.60	400.18	400.18
Diffusion time 216.3 ms	49.88	382.21	382.21	382.21	418.72	400.40	400.40
Diffusion time 316.3 ms	73.44	378.32	378.32	378.32	423.34	400.61	400.61
Diffusion time 416.3 ms	98.51	374.45	374.45	374.45	426.93	400.85	400.85

	SRM 1 year	SRM 2 years
**FF global leg**	0.39	0.41
**FF global thigh**	0.80	0.57
**cCSA global leg**	0.33	0.51
**cCSA global thigh**	0.77	0.58
**FVC**	0.54	0.45
**6MWT**	0.13	0.55
**10mWT**	0.75	0.60
**TUG**	0.0	0.67
**4S climb**	0.33	**0.87**
**4S descend**	0.18	0.42
**NSAD**	0.50	0.75
**Knee flexion**	0.41	0.52
**Knee extension**	0.06	0.44
**Ankle plantarflexion**	0.43	0.39
**Ankle dorsiflexion**	0.72	0.78

S1 Table: Standardized response mean (SRM) values for FF and cCSA in the global leg and global thigh, and for the functional and strength measurements. Values > 0.8 are marked in bold.

	age	years since diagnosis	FF	Water T2	Water T1
**FF**	0.244	**0.861****			
**Water T2**	0.351	**0.632****	**0.613****		
**Water T1**	0.187	0.514	0.528**	**0.814****	
**pH**	0.024	-0.429*	0.420**	0.318*	0.192
**FVC**	-0.013	-0.259	**-0.634****	0.302	0.039
**6MWT**	-0.202	-0.444	**-0.825****	-0.160	-0.309
**Predicted 6MWT**	-0.115	-0.441	**-0.808****	-0.131	-0.333
**10mWT**	-0.292	**-0.609****	**-0.910****	-0.176	-0.248
**TUG**	0.164	-0.005	**0.827****	**0.873****	**0.855****
**4S climb**	0.361	0.328	**0.842****	**0.789****	**0.720****
**4S descend**	0.337	0.238	**0.820****	**0.864****	**0.720****
**NSAD**	-0.322	-0.588	**-0.932****	-0.243	-0.329

S2 Table: Spearman correlation coefficients between qMRI parameters and functional measures at baseline. The qMRI outcome parameters are highlighted in light blue and the functional measures in light yellow. Strong and very strong correlations (correlation coefficient > 0.6) are marked in bold. *p<0.05, **p<0.01

	MD	FA	λ_1	λ_2	λ_3
	**Diffusion time 116.3 ms**				
**Age**	0.187	-0.106	0.231*	0.179	0.154
**Years since diagnosis**	-0.343**	0.495**	-0.152	-0.360**	-0.484**
**FF**	-0.144	0.503**	0.119	-0.190	-0.380**
**Water T2**	0.004	0.132	0.170	-0.043	-0.055
**Water T1**	-0.073	0.120	0.143	-0.074	-0.090
	**Diffusion time 216.3 ms**				
**Age**	0.201	-0.137	0.211	0.204	0.168
**Years since diagnosis**	-0.140	0.331**	0.007	-0.159	-0.293**
**FF**	0.079	0.336**	0.292**	0.026	-0.171
**Water T2**	0.183	-0.015	0.320**	0.126	0.115
**Water T1**	0.090	0.011	0.304**	0.072	0.052
	**Diffusion time 316.3 ms**				
**Age**	0.192	-0.122	0.215*	0.190	0.151
**Years since diagnosis**	-0.010	0.180	0.058	-0.018	-0.141
**FF**	0.211	0.189	0.339**	0.164	-0.014
**Water T2**	0.279*	-0.138	0.352**	0.231*	0.228*
**Water T1**	0.182	-0.095	0.340**	0.165	0.146
	**Diffusion time 416.3 ms**				
**Age**	0.193	-0.130	0.218*	0.194	0.156
**Years since diagnosis**	0.049	0.094	0.071	0.054	-0.063
**FF**	0.266*	0.106	0.350**	0.234*	0.066
**Water T2**	0.322**	-0.192	0.366**	0.276*	0.280*
**Water T1**	0.220*	-0.150	0.347**	0.208	0.196

S3 Table: Spearman correlation coefficients of the DTI parameters at each diffusion time with age, years since diagnosis, FF, water T2, and water T1. *p<0.05, **p<0.01.

		Muscle	ΔFF
**Baseline pH**		GM	-0.119
**Baseline MD**	Diffusion time: 116.3 ms	Global leg	0.018
	Diffusion time: 216.3 ms	Global leg	0.082
	Diffusion time: 316.3 ms	Global leg	0.003
	Diffusion time: 416.3 ms	Global leg	-0.138
**Baseline FA**	Diffusion time: 116.3 ms	Global leg	0.318
	Diffusion time: 216.3 ms	Global leg	0.250
	Diffusion time: 316.3 ms	Global leg	0.103
	Diffusion time: 416.3 ms	Global leg	0.262
**Baseline λ_1**	Diffusion time: 116.3 ms	Global leg	0.315
	Diffusion time: 216.3 ms	Global leg	0.253
	Diffusion time: 316.3 ms	Global leg	0.215
	Diffusion time: 416.3 ms	Global leg	0.218
**Baseline λ_2**	Diffusion time: 116.3 ms	Global leg	-0.015
	Diffusion time: 216.3 ms	Global leg	-0.082
	Diffusion time: 316.3 ms	Global leg	0.006
	Diffusion time: 416.3 ms	Global leg	-0.153
**Baseline λ_3**	Diffusion time: 116.3 ms	Global leg	-0.162
	Diffusion time: 216.3 ms	Global leg	-0.006
	Diffusion time: 316.3 ms	Global leg	-0.126
	Diffusion time: 416.3 ms	Global leg	-0.191

S4 Table: Spearman correlation coefficient between baseline pH and DTI parameters with the change in FF over 2 years (Δ).

Muscle	Baseline water T2 with ΔFF	Baseline water T1 with ΔFF
AL	**0.867****	0.524
AM	-0.536	-0.117
BF	0.117	0.500
GRA	**0.704****	0.538
RF	**0.832****	0.467
SAR	0.319	0.255
SM	**0.654****	0.503
ST	**0.825****	0.333
VI	0.339	0.510
VL	**0.521***	**0.654***
VM	**0.626***	0.441
ED	0.365	0.293
GL	0.454	0.232
GM	0.182	**0.596***
PER	0.125	0.527
SOL	0.126	0.279
TA	0.450	0.486
TP	0.033	**0.640***

S5 Table: Spearman correlations of baseline water T2 and baseline water T1 with ΔFF for the individual thigh and leg muscles. Significant correlations are marked in bold. *$p<0.05$, **$p<0.01$.

	Knee extension vs. QUAD	Knee flexion vs. HSTR	Ankle plantar flex. vs. TRIC	Ankle dorsi flex. vs. ANT
	**Absolute strength [Nm]**			
**Age**	0.016	-0.173	0.114	0.213
**Years since diagnosis**	-0.520**	**-0.686****	-0.036	-0.283
**cCSA**	**0.931****	**0.937****	0.513**	0.556**
	**Predicted strength [%]**			
**Age**	0.032	-0.050	0.311	0.173
**Years since diagnosis**	-0.456*	-0.595**	0.123	-0.327
**FF**	**-0.886****	**-0.868****	-0.461*	**-0.879****

S6 Table: Spearman correlation coefficients between the strength measurements and age, years since diagnosis, cCSA, and FF at baseline. Strong and very strong correlations (correlation coefficient > 0.6 or < -0.6) are marked in bold. *p<0.05, **p<0.01. QUAD, quadriceps; HSTR, hamstrings; TRIC, triceps surae; ANT, anterior compartment.
